# Neuroinflammatory paradigms in lysosomal storage diseases

**DOI:** 10.3389/fnins.2015.00417

**Published:** 2015-10-30

**Authors:** Megan E. Bosch, Tammy Kielian

**Affiliations:** ^1^Departments of Pharmacology and Experimental Neuroscience, University of Nebraska Medical CenterOmaha, NE, USA; ^2^Pathology and Microbiology, University of Nebraska Medical CenterOmaha, NE, USA

**Keywords:** neuroinflammation, lysosomal storage disease, microglia, astrocytes, danger-associated molecular patterns

## Abstract

Lysosomal storage diseases (LSDs) include approximately 70 distinct disorders that collectively account for 14% of all inherited metabolic diseases. LSDs are caused by mutations in various enzymes/proteins that disrupt lysosomal function, which impairs macromolecule degradation following endosome-lysosome and phagosome-lysosome fusion and autophagy, ultimately disrupting cellular homeostasis. LSDs are pathologically typified by lysosomal inclusions composed of a heterogeneous mixture of various proteins and lipids that can be found throughout the body. However, in many cases the CNS is dramatically affected, which may result from heightened neuronal vulnerability based on their post-mitotic state. Besides intrinsic neuronal defects, another emerging factor common to many LSDs is neuroinflammation, which may negatively impact neuronal survival and contribute to neurodegeneration. Microglial and astrocyte activation is a hallmark of many LSDs that affect the CNS, which often precedes and predicts regions where eventual neuron loss will occur. However, the timing, intensity, and duration of neuroinflammation may ultimately dictate the impact on CNS homeostasis. For example, a transient inflammatory response following CNS insult/injury can be neuroprotective, as glial cells attempt to remove the insult and provide trophic support to neurons. However, chronic inflammation, as seen in several LSDs, can promote neurodegeneration by creating a neurotoxic environment due to elevated levels of cytokines, chemokines, and pro-apoptotic molecules. Although neuroinflammation has been reported in several LSDs, the cellular basis and mechanisms responsible for eliciting neuroinflammatory pathways are just beginning to be defined. This review highlights the role of neuroinflammation in select LSDs and its potential contribution to neuron loss.

## Introduction: Lysosomal storage diseases

Since the discovery of the lysosome nearly 60 years ago by de Duve (1969), the field of lysosomal storage diseases (LSDs) has expanded significantly. LSDs currently encompass approximately 70 genetically distinct diseases, with a collective incidence of 1:5000 live births annually (Vitner et al., 2015). LSDs account for roughly 14% of all inherited metabolic diseases (Meikle et al., 1999; Wolf et al., 2015), among which the majority are autosomal recessive with the exception of mucopolysaccharidosis type II (MPS II) being inherited as an X-linked recessive trait (Meikle et al., 1999). A common denominator across all LSDs is lysosomal dysfunction originating from the loss of function or expression of lysosomal enzymes or other proteins that are vital for lysosomal homeostasis (Alroy et al., 2014). While the function of mutated molecules in some LSDs is well-characterized, in others, such as Niemann-Pick type C (NPC) and Juvenile Neuronal Ceroid Lipofuscinosis (JNCL), the normal function of mutated proteins is unknown (Baudry et al., 2003; Kollmann et al., 2013; Vitner et al., 2015). Nevertheless, studies using mouse models and cells from affected patients, as well as lower species when available (i.e., yeast, Drosophila), have provided valuable information concerning the downstream sequelae of LSDs where the putative function of mutated molecules remains to be identified (Pearce et al., 1999; Persaud-Sawin et al., 2002; Huang et al., 2005; Park et al., 2008; Tuxworth et al., 2011; Cotman and Staropoli, 2012).

Lysosomes are membrane bound organelles that are not only responsible for macromolecule degradation but also play a role in multiple processes required for cellular homeostasis (Appelqvist et al., 2013). This includes phagocytosis, autophagy, apoptosis, signal transduction, exocytosis, and inflammatory responses (Boustany, 2013; Alroy et al., 2014) and lysosomes harbor over 60 different enzymes responsible for lipid and protein degradation and recycling (Alroy et al., 2014). Lysosomal inclusion formation is a hallmark of LSDs, where the composition of inclusions can vary widely depending on the type of mutation to include a heterogeneous combination of sphingolipids, phospholipids, or galactosylceramide as well as proteins (Ballabio and Gieselmann, 2009). In general, most LSDs are typified by inclusions in both the CNS and periphery, since affected proteins are generally universally expressed in the lysosomes of all cells. Nevertheless, it is evident that the CNS is extremely vulnerable to impaired lysosomal function and as such, numerous LSDs are associated with neurological sequalae. LSDs are often characterized into four subgroups based on the composition of lipid accumulation, namely, mucopolysaccharidoses, glycolipidoses, mucolipidoses, and glycoproteinoses (Futerman and van Meer, 2004; Alroy et al., 2014). These glycolipids and glycoproteins comprise the main structural components of cell membranes and influence cellular trafficking, second messenger signaling, and cell-cell communication (Cooper, 2000; Farooqui, 2009). Therefore, perturbations in membrane lipid recycling or breakdown consequently lead to disruptions in myelin production, molecular transport systems, apoptosis, and immune system activation (Alroy et al., 2014).

Several LSDs exhibit neuropathological manifestations, including prominent microglial and astrocyte activation (Baudry et al., 2003; Farfel-Becker et al., 2011; Wilkinson et al., 2012; Kollmann et al., 2013). The functional implications of reactive glia in LSD progression remains unclear and can be envisioned to be either a protective response to cellular stress or detrimental, leading to the loss of critical CNS homeostatic functions. Several LSDs affecting the CNS are typified by neuroinflammation, which likely contributes to the neurodegenerative process (Cox and Cachon-Gonzalez, 2012; Rama Rao and Kielian, 2015). However, it remains unclear whether inflammatory responses are an inciting event or merely a consequence of sensing danger-associated molecular patterns (DAMPs) released from damaged/dying cells (Thundyil and Lim, 2014). This review will provide a brief overview of general aspects of neuroinflammatory paradigms in LSDs and focus on converging and diverging aspects of neuroinflammation in four LSDs with prominent CNS manifestations namely, NPC, Gaucher Disease (GD), Mucopolysaccharidoses (MPS), and the group of Neuronal Ceroid Lipofusinosis (NCL), commonly referred to as Batten Disease. Other LSDs that are typified by inflammation are briefly discussed at the conclusion, with pertinent works cited for further reading.

## Neuroinflammation in lysosomal storage diseases

Neuroinflammation is typically elicited as a protective response following CNS injury, infection, or disease; however, heightened or chronic inflammation can exert detrimental effects on neural cells (Fernandes-Alnemri et al., 2009; Lyman et al., 2014; Lee and MacLean, 2015). Inflammatory events are initiated in the CNS upon sensing DAMPs or pathogen-associated molecular patterns (PAMPs) by resident microglia and astrocytes, often leading to the mobilization of peripheral leukocytes into the CNS. The goal is a swift response to contain the insult, which is rapidly downregulated to avoid unnecessary bystander damage (Lyman et al., 2014). This process is facilitated by several factors, including inflammatory cytokines and chemokines, trophic factors, phagocytosis, and regulated forms of cell death (i.e., apoptosis or pyroptosis) (Bergsbaken et al., 2009; Lyman et al., 2014; Heneka et al., 2015). However, persistent inflammation can elicit additional damage, further perpetuating the inflammatory cycle and CNS pathology.

Microglia are the principal neuroinflammatory cells in the CNS parenchyma, which constantly survey for stress signals (i.e., DAMPs), phagocytose cellular debris, and provide trophic support (Rock et al., 2004; Heneka et al., 2015). Microglia sense DAMPs released from damaged or dying neurons (Thundyil and Lim, 2014) via Toll-like receptors and other pattern recognition receptors (Hanke and Kielian, 2011; Lyman et al., 2014). Once activated, microglia are a major source of chemokines and cytokines that serve to recruit and activate peripheral immune cells at affected sites, respectively (Amor et al., 2014). In addition, these mediators can act in an autocrine/paracrine manner to activate neighboring microglia, astrocytes, neurons, or oligodendrocytes. The consequences of cytokine action vary between each cell type and can include perpetuating inflammatory mediator release, altering target cell function, or inducing cell death (Vezzani and Viviani, 2015).

Astrocytes are the most abundant cell type in the brain and provide neurotrophic support, synchronize neurotransmitter metabolism and release, and regulate the extracellular milieu (Haydon and Nedergaard, 2015; Verkhratsky et al., 2015; Verkhratsky and Parpura, 2015). Besides these critical homeostatic functions, astrocytes also participate in neuroinflammatory responses through their robust secretion of various chemokines (Dong and Benveniste, 2001; Farina et al., 2007). Upon activation, astrocytes undergo a morphological transformation typified by increased intermediate filament expression (i.e., glial fibrillary acidic protein (GFAP) and vimentin) and reactive astrocytes have been implicated in the pathogenesis of various neurodegenerative diseases (van der Hel et al., 2005; Kulijewicz-Nawrot et al., 2013; Assous et al., 2014). For example, activated astrocytes can produce chemokines, reactive oxygen/nitrogen species, and various toxic molecules. In addition, astrocyte activation often compromises glutamate homeostatic pathways, such as glutamate uptake and recycling mediated by glutamate transporters and glutamine synthetase, respectively (Kulijewicz-Nawrot et al., 2013; Heneka et al., 2015). Reactive astrocytes have been detected during the acute phase of disease in animal models of Alzheimer's Disease (AD), Parkinson's Disease (PD), and several LSDs, likely setting the stage for neuronal dysfunction and neurodegeneration (DiRosario et al., 2009; Farfel-Becker et al., 2011; Heneka et al., 2015; Lee and MacLean, 2015). In response to insult or injury, reactive astrocytes have been shown to provide a bordering function to isolate areas of damage by forming a “glial scar” that prevents dying cells from impacting healthy tissue. Neuroinflammation also elicits astrocyte hemichannel opening concomitant with reduced gap junction activity, which together can significantly disrupt neurotransmitter regulation (Karpuk et al., 2011; Orellana et al., 2011). However, chronic neuroinflammation can also impair astrocyte neurotrophic support, making neurons more vulnerable to cytotoxic events (Lee and MacLean, 2015).

Neuroinflammation has been reported in several LSDs, although the intensity of inflammatory changes and the molecular pathways responsible for triggering neuroinflammation in each likely differ. Although LSDs are caused by distinct genetic mutations they share some common downstream attributes, the foremost being lysosomal storage material accumulation. While the biochemical composition of storage material differs between various LSDs, emerging evidence suggests that it can either activate or perpetuate neuroinflammation, which can contribute to neuronal death (Platt et al., 2012). Indeed, recent studies report that an imbalance between the synthesis and degradation of sphingolipid intermediates plays a prominent role in eliciting neuroinflammatory outcomes (Nixon, 2009). For example, TNF-α can activate sphingomyelinase (SMase), a key enzyme responsible for ceramide production, which leads to elevated ceramide levels that have been reported in JNCL (Puranam et al., 1997; Mencarelli and Martinez-Martinez, 2013). It is well appreciated that high ceramide concentrations can also perpetuate inflammatory cascades such as NF-κB activation, trigger inflammasome activation, and disrupt normal cellular functions (Nixon, 2009; Mencarelli and Martinez-Martinez, 2013). Ceramide accumulation correlates with decreases in sphinosine-1-phosphate (S1P), thereby inducing apoptotic pathways and glial activation (Nixon, 2009). The S1P antagonist FTY720 has been shown to attenuate glial activation and neuroinflammatory processes, which leads to reduced relapse rates in multiple sclerosis (Davies et al., 2013; Brunkhorst et al., 2014), although its effects in LSDs remain to be investigated. Glucosylceramide accumulation in Gaucher disease (GD), resulting from glucocerebrosidase deficiency, has been shown to augment cytokine production in microglia (Hong et al., 2006). Likewise, glucocerebrosidase loss in a mouse neuropathy model of GD causes microglial activation as early as postnatal day 12 (Farfel-Becker et al., 2011). The loss of lysosomal function coupled with lipid inclusion formation disrupts lysosomal membrane integrity, resulting in increased permeability and translocation of lysosomal contents into the cytosol (Pereira et al., 2010). For example, defective lysosomes release hydrolases, metabolites, and cathepsins into the cytoplasm that can be sensed as intracellular DAMPs (Futerman and van Meer, 2004). Cathepsins, in particular cathepsin B, have been shown to trigger NLRP3 inflammasome activation that contributes to neuroinflammation as well as induces neuronal apoptosis (Tschopp and Schroder, 2010). Additional intracellular changes that have been reported in various LSDs also have the ability to trigger and/or propagate neuroinflammation. For example, several LSDs display elevated intracellular Ca^2+^ levels (Vitner et al., 2010; Boustany, 2013) and disruption of Ca^2+^ homeostasis can induce multiple inflammatory processes in many CNS cell types (Zundorf and Reiser, 2011). In the case of microglia, elevated Ca^2+^_*i*_ can induce inflammasome activity, cytokine release, and NF-κB activation. In neurons, increased Ca^2+^_*i*_ disrupts synchronized synaptic activity, and dysregulated neuronal firing can augment glutamate and ATP release as well as other “danger” signals that, in turn, trigger Ca^2+^_*i*_ elevations in surrounding glia (Sama and Norris, 2013), effectively perpetuating the response. In addition, storage material accumulation in LSDs disrupts normal ER function and causes ER stress, eliciting an unfolded protein response (UPR), a physiological reaction that halts protein synthesis in an effort to correct disrupted protein folding. However, a persistent UPR induces apoptosis by activating various signaling cascades, which can have catastrophic effects on the CNS (Bronson et al., 1993; Doyle et al., 2011; Vitner and Futerman, 2013). Besides inducing cell death, ER stress can also trigger intracellular proinflammatory processes (Salminen et al., 2009). For example, Ca^2+^ released from intracellular stores leads to NF-κB activation and subsequent cytokine, chemokine, and ROS production, which can exacerbate neuropathology (Pahl and Baeuerle, 1997; Salminen et al., 2009). Additionally, ER stress in astrocytes can trigger the activation of surrounding microglia, leading to increased IL-6 production (Meares et al., 2014). Neuroinflammation is an overarching theme in several LSDs that may propagate neurodegeneration. The following sections will discuss neuroinflammatory events in select LSDs and the impact of neuroinflammation on neuron loss in these disorders.

## Niemann-pick type C (NPC)

Described in the late 1920s by Albert Niemann and Ludwig Pick, NPC is caused by a mutation in either NPC1 (95% incidence) or NPC2 (5% incidence), which manifest as similar disorders (Williams et al., 2014; Alobaidy, 2015). The frequency of NPC has been estimated at 1:120,000 live births annually (Alobaidy, 2015). There are four age-dependent variants of NPC namely, infantile, late infantile, juvenile, and adult. The infantile and late infantile forms are most severe and occur between 2 months to 6 years of age and are typified by cerebellar ataxia, dementia, cognitive decline, seizures, and premature death (Vanier, 2010). NPC1 encodes for a lysosomal/endosomal transmembrane protein of unknown function, while NPC2 produces a soluble mannose-6-phosphorylated lysosomal transport protein (Williams et al., 2014). Both NPC1 and NPC2 regulate the intracellular trafficking and processing of cholesterol and other lipids (Baudry et al., 2003; Gallala et al., 2011). Mutation in either NPC1 or NPC2 results in the lysosomal accumulation of cholesterol and sphingolipids, in particular glycosphingolipids and sphingomyelin (Rosenbaum and Maxfield, 2011). The pathogenic mechanisms responsible for NPC disease remain to be identified.

Microglial activation has been shown to occur early in NPC^−/−^ mice and precedes neurodegeneration. Multiple brain regions in NPC ^−/−^ mice display increased levels of TNF-α and IL-1β as well as heightened apoptosis. These areas correspond to brain regions with profound neuron loss at later stages of the disease (Wang et al., 2010; Peake et al., 2011). Baudry et al. found that microglial activation was significantly increased in the striatum, thalamus, substantia nigra pars compacta, neocortex, and cerebellum of NPC ^−/−^ mice beginning as early as postnatal week 2 (Baudry et al., 2003). Areas of microglial activation correlated with the onset of axonal damage by postnatal week 4 and by week 10, almost all Purkinje cells within the cerebellum were lost (Baudry et al., 2003). Neuronal deterioration in NPC has a specific spatial and temporal configuration, beginning with proximal sensory and motor projection loss followed by neuron soma dropout (Pressey et al., 2012; Vitner et al., 2015). Compared to microglia, astrocyte activation is delayed in NPC until 4–10 weeks of age and is likely elicited in response to neuronal damage. It has been suggested that astrocyte and microglial activation in NPC is a protective event designed to phagocytose damaged neurons to limit subsequent DAMP release (Minghetti and Levi, 1998; Baudry et al., 2003). However, whether neuronal death that occurs during NPC is cell-autonomous or non-cell autonomous is not completely understood (Baudry et al., 2003; Peake et al., 2011).

Neuroinflammation has been suggested to play an important role in NPC, since several studies have shown that disease progression can be delayed with anti-inflammatory compounds (Smith et al., 2009; Williams et al., 2014). Specifically, treatment with non-steroidal anti-inflammatory drugs (NSAIDs) in combination with miglustat and the antioxidant curcumin has been shown to reduce microglial activation and Purkinje cell death, effectively extending the survival of NPC^−/−^ mice (Williams et al., 2014). Recent studies have shown that directly targeting cholesterol accumulation can also reduce neurological damage and slow NPC progression. Specifically, 2-hydroxypropyl-β-cylodextrin (HPβCD) reduced cholesterol and ganglioside accumulation in the cerebellum and enhanced Purkinje cell survival, which resulted in increased life span in both mouse and feline NPC models (Camargo et al., 2001; Vite et al., 2015). HPβCD also reduced CD68 (microglia/macrophage) and GFAP (astrocyte) expression in the brains of NPC^−/−^ mice following a 10 week treatment period (Alam et al., 2014). These studies strongly suggest that neuroinflammation is a major factor in NPC progression and support the concept that targeting neuroinflammation may potentially extend the lives of patients with NPC, although this possibility remains speculative at the present time. Due to the multifaceted nature of NPC, a combinational therapy targeting inappropriate inflammation along with reversing cholesterol accumulation will likely be required to maximally impact disease progression. This approach is also likely necessary for other LSDs that have additional pathological sequelae besides lysosomal storage accumulation.

## Gaucher disease (GD)

Gaucher disease (GD) is the most common LSD arising from a mutation in the lysosomal enzyme glucosylceramidase (GlcCerase), which causes a decrease or complete loss of enzymatic activity and intracellular glucosylceramide (GlcCer) accumulation. The incidence of GD is 1 out of every 40,000–60,000 live births a year and is classified into three age-dependent forms. Type 1 is the most common (90% of patients) and is typified by splenomegaly, hepatomegaly, anemia, bone pain, and skeletal lesions (Vitner and Futerman, 2013). Type I GD is not associated with any neurological symptoms, whereas Types 2 and 3 display prominent neuropathology. Type 2 GD is extremely rare and manifests around 6 months of age with a life expectancy of 2–4 years, whereas Type 3 presents with similar neurological abnormalities as Type 2 but at a later interval in young adolescence (Sidransky et al., 1996; Vitner and Futerman, 2013). The relationship between GlcCer accumulation and progression of neuropathological sequelae in GD is not completely understood (Vitner and Futerman, 2013).

GD Types 2 and 3 have been linked to elevated proinflammatory cytokine (IL-1α, IL-1β, TNF-α, IL-6) and reactive oxygen species (ROS) production in the brains of *gba* null mice and blood of GD patients (Barak et al., 1999; Hong et al., 2006; Vitner and Futerman, 2013). GlcCer accumulation within inflammatory cells, macrophages and natural killer cells in particular, impairs cell migration, antigen presentation, and killing functions that contribute to persistent and altered inflammatory responses in GD (Bettman et al., 2015; Nair et al., 2015). Early studies examining the impact of neuroinflammation on GD progression were limited by the fact that the *gba* knockout (KO) mouse model has an exceptionally short life span of 1 day (Tybulewicz et al., 1992). Recently, Enquist et al. created a Gba^flox/flox^ nestin-Cre mouse that deletes GlcCerase in neural progenitor cells that give rise to astrocytes and oligodendrocytes, leaving the enzyme fully functional in microglia which are derived from the yolk sac (Alliot et al., 1999; Enquist et al., 2007). These studies revealed the early synthesis of anti-inflammatory cytokines, which later shifted to a pro-inflammatory phenotype (Enquist et al., 2007; Vitner et al., 2012), suggesting that neuroinflammation may be elicited by DAMPs released from diseased neurons, astrocytes, and/or oligodendrocytes. By extension, these DAMPs are expected to trigger robust microglial proinflammatory activity, culminating in a neurotoxic environment that promotes neurodegeneration. In addition, studies have shown that GlcCer accumulation is linked to disrupted Ca^2+^ homeostasis in neurons, where GlcCer interacts with the ryanodine receptor, which is responsible for ER Ca^2+^ release (Korkotian et al., 1999; Vitner and Futerman, 2013). Elevated Ca^2+^_*i*_ levels in neurons increases sensitivity to neurotoxic agents and induces DAMP release (Sama and Norris, 2013). Unfortunately, anti-inflammatory compounds have had no effect on prolonging survival of Gba^−/−^ mice (Vitner et al., 2012). These findings suggest that neuronal loss may primarily be cell-autonomous and that therapeutic targeting should be more focused on the contribution of neurons to inflammatory reactions.

## Mucpolysaccharidosis (MPS)

MPS encompasses a heterogeneous group of LSDs that result from a deficiency in lysosomal enzymes responsible for glycosaminoglycan (GAG) degradation (Wraith, 1995; Archer et al., 2014). MPS forms represent about 30% of all LSDs, with an incidence in 1 out of every 25,000 births (2011). Clinical symptoms typically present early in life with either severe or attenuated phenotypes (Muenzer, 2004). Patients can experience neurodegeneration, behavioral deficits, such as hyperactivity and aggressiveness, and peripheral organ dysfunction (Archer et al., 2014). MPS forms are caused by a mutation in any one of 11 distinct hydrolases that function in GAG degradation (Alroy et al., 2014). The loss of catabolic function causes an accumulation of heparan sulfate (HS) and dermatan sulfate in lysosomes followed by subsequent ganglioside accumulation in the CNS, similar to what is found in GM-gangliosidosis (Archer et al., 2014). Ganglioside accumulation has been shown to disrupt lysosomal function, leading to multi-organ failure and death (Archer et al., 2014).

Astrocyte and microglial activation has been suggested to impact MPS pathogenesis (Campos and Monaga, 2012; Archer et al., 2014). As storage material accumulates within the lysosome it can disrupt lysosomal membrane integrity, leading to lysosomal membrane permeability and subsequent release of HS, gangliosides, and cysteine proteases into the cytoplasm (Pereira et al., 2010). In turn, these molecules can act as intracellular DAMPs to perpetuate microglial activation and promote inflammatory responses (Campos and Monaga, 2012). An alternative possibility is that HS directly activates Toll-like receptor 4 (TLR4) that elicits pro-inflammatory cytokine and reactive oxygen species (ROS) production to perpetuate the inflammatory process. However, in this instance, HS would need to be released into the extracellular milieu to engage TLR4, which appears only plausible via cell death or lysosomal exocytosis. Nevertheless, inflammatory responses elicited by the release of various DAMPs from cellular injury likely contribute to the neurodegenerative process in MPS.

Besides microglial responses, another important factor contributing to inflammation in MPS is peripheral immune system activation (DiRosario et al., 2009). In multiple models of MPS (MPS I, IIIA, IIIB) numerous chemokines are elevated in the brain, including CCL3 and CCL4, which correlate with peripheral T cell and macrophage infiltrates (Villani et al., 2007; Arfi et al., 2011). Additionally, treatment of MPS IIIA mice with acetylsalicylic acid over a 6 month period significantly attenuated cytokine and chemokine expression, apoptosis-related genes, and increased the antioxidant status of the brain as demonstrated by increased glutathione levels (Arfi et al., 2011). Another report demonstrated that immunosuppression with prednisolone significantly slowed CNS disease progression in a mouse model of MPS IIIB (DiRosario et al., 2009). Collectively, these findings suggest that anti-inflammatory drugs dampen neuroinflammation and could potentially improve disease outcome in specific MPS forms (Arfi et al., 2011).

## Neuronal ceroid lipofuscinosis (NCL)

NCLs are a diverse group of autosomal recessively inherited LSDs caused by a mutation in one of 14 different ceroid lipofuscinosis (CLN) genes (Kollmann et al., 2013). NCLs have been estimated to occur in 1 out of every 100,000 live births per year (Lerner et al., 1995; Jalanko and Braulke, 2009; Santorelli et al., 2013). There are four age-dependent classifications of NCL that correlate with specific genetic mutations; namely, infantile (INCL), late infantile (LINCL), juvenile (JNCL), and adult onset (Cooper, 2003). The four subtypes generally present with similar symptoms that include visual deterioration, neurocognitive and physical decline, seizures, and premature death, although the sequence of symptom appearance differs between forms (Mitchison et al., 2004; Finn et al., 2011; Cotman and Staropoli, 2012; Anderson et al., 2013). The early onset NCLs, including infantile and late infantile, are the most aggressive in terms of the short interval from diagnosis to death and are generally typified by more prominent neuroinflammatory responses (Dolisca et al., 2013). The various NCL-related mutations result in either a loss in lysosomal enzymes, lysosomal transmembrane proteins, or synaptic vesicle-associated proteins (Kollmann et al., 2013). Although the function of some NCL-related genes are known (i.e., CLN1 and CLN2 encode for the lysosomal enzymes PPT-1 and TPP-1, respectively), others remain elusive (i.e., CLN3, CLN5, CLN6). Mutations in each of the CLN proteins lead to the accumulation of lysosomal storage material whose biochemical composition varies according to each disease type. For example, storage material can contain mitochondrial ATP synthase subunit c, sphingolipids, and lipofuscin, the latter of which is responsible for the autofluorescent properties of storage material (Haltia, 2003; Williams et al., 2006). To date there is no cure for any of the NCL forms, only treatments to manage clinical symptoms.

**Figure 1:**
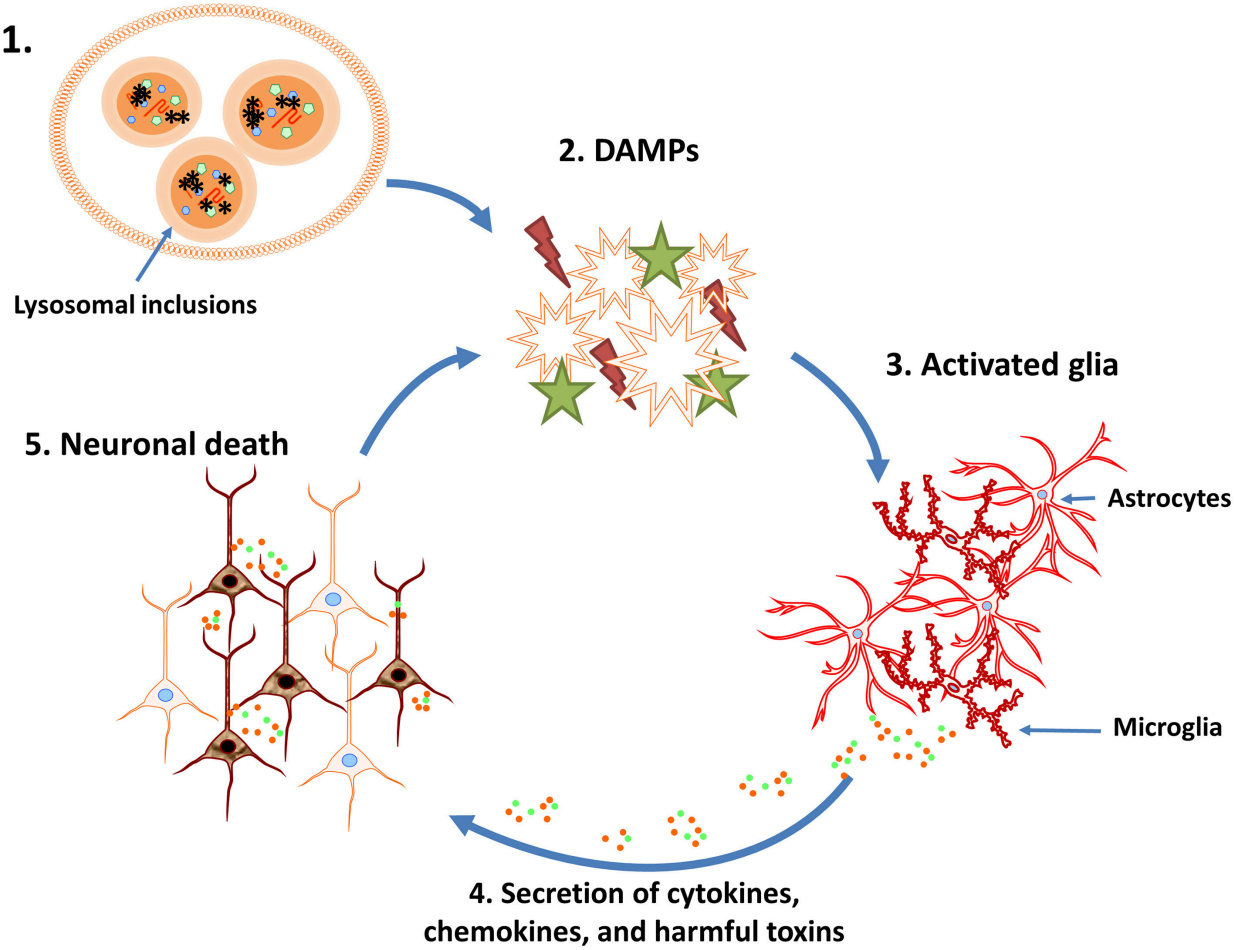
**Potential vicious cycle that perpetuates neuronal loss during lysosomal storage diseases (LSDs)**. 1. Neuroinflammation associated with LSDs may be initiated, in part, by the disruption of normal lysosomal function and accumulation of heterogeneous inclusions. 2. Lysosome dysfunction triggers the activation and/or release of danger associated molecular patterns (DAMPs). 3. DAMPs activate surrounding glial cells, resulting in their proliferation and initiating inflammatory signaling pathways. 4. Activated glia produce chemokines, cytokines, and other inflammatory mediators (i.e., reactive oxygen/nitrogen intermediates). 5. The cytotoxic environment and loss of glial support results in neuron death and DAMP release, which further perpetuates the neuroinflammatory cycle.

Emerging evidence suggests that neuroinflammation may play a role in NCL pathogenesis, although the timing and intensity of inflammatory events in each disease type likely differ. For example, neuroinflammation is robust in the most aggressive form of NCL (INCL), where glial activation parallels increased proinflammatory cytokine production and peripheral immune cell infiltration into the brain (Macauley et al., 2012). Microglial and astrocyte activation has also been shown to precede neuronal death in affected brain regions in JNCL (Pontikis et al., 2004). Primary microglia isolated from CLN3 mutant (CLN3^Δex7/8^) mice exist in a primed pro-inflammatory state (Xiong and Kielian, 2013), producing heightened levels of numerous mediators, including TNF-α, IL-9, IL-1β, IL-1α, IL-15, and IL-10 following exposure to DAMPs elevated in the JNCL brain (Puranam et al., 1997; Mencarelli and Martinez-Martinez, 2013). As mentioned above, the degree of neuroinflammation in the various NCL forms appears to correlate with the severity of disease progression. For example, INCL and LINCL, which are more aggressive in their clinical course compared to JNCL (Hawkins-Salsbury et al., 2013), display more rampant neuroinflammation (Palmer et al., 2013). This could be explained by the compressed time frame of disease typified by extensive and rapid neuronal death (Kielar et al., 2007). This would effectively increase DAMP release and trigger early glial activation that would impact neuronal function in a non-cell autonomous manner, causing direct inflammatory damage and loss of trophic support, which would further exacerbate neuronal death in a vicious feedback loop (Figure 1). In fact, multiple NCL forms are typified by synaptic loss coupled with an imbalance in critical neurotransmitter functions (Palmer et al., 2013). For example, patients with CLN1, CLN2, and CLN6 mutations exhibit decreased GABA and glutamate levels, while individuals with CLN3 mutations have excessive glutamate and lower GABA in the CNS (Sitter et al., 2004; Pears et al., 2005, 2007). The reasons responsible for differential neurotransmitter dysfunction between various NCL forms are not understood; however, it is clear that neurotransmitter imbalances likely exacerbate defects in neuronal function that are observed in these diseases. In addition, loss of microglial and astrocyte homeostatic functions increases neuron vulnerability to not only toxic inflammatory molecules but also insults from surrounding neurons, such as neurotransmitter excitotoxicity (Hachiya et al., 2006). A role for neuroinflammation in NCL progression is only beginning to be mechanistically explored, with a recent study showing beneficial effects of anti-inflammatory treatments (Macauley et al., 2012). For example, Macauley et al. found that a combination of anti-inflammatory compounds increased the survival rate of Ppt1^−/−^ mice, a model of INCL, concomitant with reduced cytokine production and less regional brain atrophy. This study suggests that anti-inflammatory treatment can be beneficial in slowing neurodegeneration caused by inflammatory insults (Macauley et al., 2012); however, it remains to be determined whether these compounds exert their beneficial effects by limiting inflammation or rather, also impact other disease attributes of neurons and glia.

## Nflammation in other lysosomal storage diseases

In addition to the LSDs described in detail above, there is emerging evidence implicating neuroinflammation in other LSDs, including Tay-Sachs, Sandhoff disease, and Fabry disease. Tay-Sachs and Sandhoff disease are both GM2 gangliosidoses, typified by ganglioside accumulation throughout the CNS (Jeyakumar et al., 2003). The mechanisms responsible for disease progression and cell death in these LSDs remains unknown; however, murine models and patient samples suggest a role for inflammation. Neuroinflammation has been found to precede symptom onset in mouse models of Tay-Sachs and Sandhoff disease (Jeyakumar et al., 2003). In addition, inflammatory mediators are elevated in patients with Tay-Sachs or infantile gangliosidosis, including MCP-1α, MIP-1β, and TNFR2, and the intensity of inflammation is heightened in the more aggressive infantile forms compared to the juvenile forms (Hayase et al., 2010; Utz et al., 2015). This pattern is similar to what has been reported in multiple LSDs (Pahl and Baeuerle, 1997; Vanier, 2010; Hawkins-Salsbury et al., 2013). Fabry disease is the second most common LSD and results from a reduction or loss of α-galactosidase A, leading to the accumulation of neutral glycosphingolipids (Meikle et al., 1999; Mauhin et al., 2015). Patients with Fabry disease have elevated levels of IL-6, IL-1β, and TNF-α in the systemic circulation, revealing an inflammatory aspect of this LSD (De Francesco et al., 2013; Mauhin et al., 2015). When taken together, neuroinflammation has been implicated as a pathological hallmark of a wide range LSDs, suggesting it may be a viable target when considering potential new therapeutics.

## Outstanding questions and future research avenues

Although our understanding of LSDs has significantly expanded since the discovery of the lysosome by DeDuve in the 1950s, many questions remain unanswered. There is currently no cure for any of the LSDs mentioned and the only available therapeutics target disease symptoms. Therefore, it is imperative to identify novel therapeutics to not only alleviate symptoms but also slow disease progression and extend life expectancy. The genetic mutations unique to each LSD result in distinct malfunctions of a specific lysosomal mechanism. By extension, this would suggest that each disease would present with different symptoms, neuropathology, and disease progression. However, many similarities exist between these attributes among the diverse array of LSDs, which suggests the possibility of unifying mechanisms of downstream pathology. If accurate, in-depth comparisons of the similarities and differences between LSDs have the potential to direct researchers toward overarching mechanistic pathways to target for treatment.

One of the main attributes shared across several LSDs is neuropathological symptoms, such as seizures and neurocognitive decline, leading to premature death (Muenzer, 2004; Lim et al., 2006; Vanier, 2010; Anderson et al., 2013; Vitner and Futerman, 2013). Substrate accumulation within the lysosomes of neurons is expected to disrupt their normal homeostatic functions, although it is clear that the presence of inclusion material *per se* is not a requisite for death, since only neurons in specific brain regions are lost in several LSDs (Cotman et al., 2002; Baudry et al., 2003; Muenzer, 2004). Impaired neuronal function can augment glutamate and ATP release, which can be sensed by surrounding glia as danger signals triggering their activation to clear senescent/damaged cells or debris in an attempt to restore homeostasis, along with offering trophic support to surrounding neurons. Glial cells also produce trophic factors, such as TGF-β and matricellular proteins in an effort to halt damage and promote neuronal survival and health (Dheen et al., 2007); however, sustained DAMP release can result in chronic pathological inflammation. A role for inflammation is supported by the increase in glial activation that precedes neurodegenerative changes in NPC, GD, MPS, and NCLs (Baudry et al., 2003; Pontikis et al., 2004; Farfel-Becker et al., 2011; Archer et al., 2014) and the positive outcomes of anti-inflammatory therapies on extending the survival of NPC and MPS mouse models (Arfi et al., 2011; Williams et al., 2014).

There is a strong correlation between neuroinflammation and disease progression in several LSDs; however it is unclear whether neuroinflammation is an inciting factor or a response to neuronal damage. However, glial activation and neuroinflammation has been shown to precede the onset of neuron loss in many LSDs, which strongly implies that neuroinflammation contributes to neurodegeneration. This is further supported by several reports, as noted in previous sections, that anti-inflammatory agents are capable of mitigating disease progression and improving neuronal survival. Besides the contribution of glial cells, neurons are also capable of releasing DAMPs to signal neuroinflammation (Thundyil and Lim, 2014). In the case of JNCL, inclusions are primarily observed in neurons throughout multiple brain regions (Cotman et al., 2002; Mole et al., 2005; Burkovetskaya et al., 2014). Additional evidence to support neuronal induction of inflammation is provided by a GD mouse model where GlcCerase was ablated in neurons, astrocytes, and oligodendrocytes, leaving microglia with a fully functional GlcCerase. These mice exhibited both microglial and astrocyte activation by postnatal day 12 (Farfel-Becker et al., 2011). These studies have also shown glucosylceramide accumulation primarily in cortical and thalamic neurons but not glia (Farfel-Becker et al., 2011), suggesting that neurons have to potential to direct inflammatory responses in LSDs and perpetuate disease progression.

There is currently no cure for any of the LSDs. For those that are caused by the lack of enzymatic activities, current therapeutic approaches include enzyme replacement therapy, which is costly and presents many challenges to achieve therapeutic enzyme levels in the brain because the blood-brain barrier remains intact in many instances (Bennett and Mohan, 2013). Therefore, new approaches, such as gene therapy, should be considered in an effort to correct the mutation and extend the survival of patients afflicted with LSDs. A better understanding of the cellular basis of neuroinflammation as well as how neuroinflammatory mechanisms contribute to LSD pathogenesis may enable combinational therapeutic approaches to exert a positive impact on disease outcome. Indeed, this approach has been successfully employed in Krabbe Disease, another LSD with robust CNS manifestations, where combined gene therapy and bone marrow transplantation produces a synergistic effect on survival compared to either therapeutic alone (Escolar et al., 2005; Hawkins-Salsbury et al., 2011, 2015; Qin et al., 2012). However, bone marrow transplantation has shown limited efficacy in other LSDs, again highlighting the heterogeneity between different disorders and need to tailor potential therapeutics accordingly.

## Funding

This work was supported by the National Institute of Neurological Disorders and Stroke (NINDS) 1R21NS084392-01A1 to TK and a UNMC Graduate Fellowship to MB.

### Conflict of interest statement

The authors declare that the research was conducted in the absence of any commercial or financial relationships that could be construed as a potential conflict of interest.
